# Supplementary material for: mlr3mbo: Bayesian optimization in R
Source: Patterns (N Y). 2026 Jul 21;7(10):101614. doi: 10.1016/j.patter.2026.101614 (PMC13674303; doi:10.1016/j.patter.2026.101614)
Supplement: Supplementary file 1 [file mmc1.pdf]

Patterns, Volume *7*

Supplemental information

mlr3mbo: Bayesian optimization in R

Marc Becker, Lennart Schneider, Martin Binder, Lars Kotthoff, and Bernd Bischl

Table S1. Software packages referenced in this paper. Versions listed are the latest releases available at the time of writing.

Package	Version	Language	Source
mlrMBO	1.1.6	R	CRAN
mlr3	1.6.0	R	CRAN
mlr3tuning	1.6.0	R	CRAN
mlr3pipelines	0.11.0	R	CRAN
mlr3learners	0.14.0	R	CRAN
mlr3verse	0.3.1	R	CRAN
bbotk	1.10.0	R	CRAN
paradox	1.0.1	R	CRAN
ranger	0.18.0	R	CRAN
nloptr	2.2.1	R	CRAN
data.table	1.18.2.1	R	CRAN
future	1.70.0	R	CRAN
mirai	2.6.1	R	CRAN
rush	1.1.0	R	CRAN
DiceOptim	2.1.2	R	CRAN
GPareto	1.1.9	R	CRAN
rBayesianOptimization	1.2.2	R	CRAN
ParBayesianOptimization	1.2.6	R	CRAN (archived)
tune	2.1.0	R	CRAN
tidyverse	2.0.0	R	CRAN
SMAC3	2.4.0	Python	PyPI
HEBO	0.3.6	Python	PyPI
Ax	1.2.4	Python	PyPI
BoTorch	0.17.2	Python	PyPI
Optuna	4.8.0	Python	PyPI
Hyperopt	0.2.7	Python	PyPI
Dragonfly	0.1.7	Python	PyPI
NUBO	1.2.1	Python	PyPI
BoFire	0.3.1	Python	PyPI
BayBE	0.14.3	Python	PyPI
BOA	0.11.0	Python	PyPI
BayesOpt	0.8	C++	GitHub
BayesianOptimization.jl	0.2.5	Julia	GitHub
bayesopt	R2025a	MATLAB	Built-in toolbox

Table S2. Acquisition functions. Acquisition functions are constructed with the `acqf(key)`. Call `$help()` to access the help page of the acquisition function e.g. `acqf("ei")$help()`. The help page describes the formula of the acquisition function and the parameters that can be set.

Key	Description	Reference
aei	Augmented expected improvement for noisy optimization. c controls the exploration-exploitation trade-off	Huang et al. [S1]
cb	Lower / upper confidence bound. λ controls the exploration-exploitation trade-off	Snoek et al. [S2]
ehvi	Expected hypervolume improvement for multi-criteria optimization	Emmerich et al. [S3]
ehvigh	Expected hypervolume improvement via GH quadrature for multi-criteria optimization	Rahat et al. [S4]
ei	Expected improvement	Jones et al. [S5]
ei_log	Expected improvement. Assumes that the surrogate models on log scale	Hutter et al. [S6]Watanabe [S7]
eips	Expected improvement per second. Balances performance and computation time	Snoek et al. [S2]
mean	Posterior mean. Mean of the surrogate model	
multi	Multiple acquisition functions. Combines multiple acquisition functions	
pi	Probability of improvement.	Kushner [S8]
sd	Posterior standard deviation. Standard deviation of the surrogate model	
smsego	S-metric selection evolutionary global optimization for multi-criteria optimization. Maximizing the expected improvement in the hypervolume	Beume et al. [S9]
stochastic_cb	Stochastic lower / upper confidence bound for asynchronous optimization. Samples λ from a distribution	Snoek et al. [S2]
stochastic_ei	Stochastic expected improvement for asynchronous optimization. Samples ϵ from a distribution	Jones et al. [S5]

Table S3. Acquisition function optimizers. Optimizers are constructed with the `acqo(key)`. Call `$help()` to access the help page of the acquisition function optimizer e.g. `acqo("direct")$help()`. The help page describes the parameters that can be set.

Key	Search space type	Default settings	Reference
direct	Numeric	$5 \cdot d$ restarts and $100 \cdot d^2$ function evaluations	Jones et al. [S10]
lbfgs	Numeric	$5 \cdot d$ restarts and $100 \cdot d^2$ function evaluations	Byrd et al. [S11]
cmaes	Numeric	ABIPOP with unlimited restarts and $100 \cdot d^2$ function evaluations	Hansen and Ostermeier [S12]
random_search	Mixed and dependencies	$100 \cdot d^2$ iterations	
local_search	Mixed and dependencies	10 neighbors and $100 \cdot d^2$ iterations	Hutter et al. [S6]

Table S4. `Terminator` classes in the `bbotk` package. Terminators are constructed with the `trm(key)` function. Call `$help()` to access the help page of the terminator e.g. `trm("stagnation")$help()`. The help page describes the parameters that can be set.

Key	Description
clock_time	Clock time exceeds a given limit
combo	A combination of terminators is met
evals	Number of evaluations exceeds a given limit
none	Optimization is finished
perf_reached	Performance reaches a given level
run_time	Run time exceeds a given limit
stagnation	Performance has not improved for a given number of iterations
stagnation_batch	Performance has not improved for a given number of batches
stagnation_hypervolume	Hypervolume stagnates

Table S5. Search space for purely numeric instances.

Parameter	Values	Dependency	Default
Input trafo	None, unitcube	–	None
Output trafo	None, standardize, log	–	None
Init	Random, LHS, Sobol	–	Random
Init size	0.05, 0.10, 0.25	–	0.25
Random interleave	0, 2, 4	–	0
Surrogate	RF, GP	–	GP
Extratrees	TRUE, FALSE	Surrogate = RF	FALSE
Trees	10, 500	Surrogate = RF	500
Variance estimator	Jackknife, ESD, LTV	Surrogate = RF	Jack
Kernel	Gauss, Matérn $3/2$, Matérn $5/2$, Exp	Surrogate = GP	Gauss
Nugget	0, 1e-3, 1e-8	Surrogate = GP	0
Scaling	TRUE, FALSE	Surrogate = GP	FALSE
Acquisition function	EI, LCB, PI, mean	–	EI
λ	1, 3, 10	Acqf = LCB	1
Optimizer	RS 1000, RS, LS, DIRECT, CMA-ES, L-BFGS-B	–	RS 1000
ϵ decay	TRUE, FALSE	Acqf = EI	FALSE
λ decay	TRUE, FALSE	Acqf = LCB	FALSE

Table S6. Search space for mixed-hierarchical instances.

Parameter	Values	Dependency	Default
Input trafo	None, unitcube	–	None
Output trafo	None, standardize, log	–	None
Init	Random, LHS, Sobol	–	Random
Init size	0.05, 0.10, 0.25	–	0.25
Random interleave	0, 2, 4	–	0
Variance estimator	Jackknife, ESD, LTV	–	ESD
Trees	10, 500	–	500
Acquisition function	EI, LCB, PI, mean	–	EI
λ	1, 3, 10	Acqf = LCB	1
Optimizer	RS 1000, RS, FS, LS	–	RS 1000
ϵ decay	TRUE, FALSE	Acqf = EI	FALSE
λ decay	TRUE, FALSE	Acqf = LCB	FALSE

Table S7. YAHPO-SO v1 numeric instances. Each row lists the scenario, instance identifier, underlying data set, and search space dimension.

Scenario	Instance	Data set	Dimension
lcbench	189873	dionis	7
lcbench	189906	segment	7
lcbench	167168	vehicle	7
rbv2_rpart	14	mfeat-fourier	5
rbv2_rpart	40499	texture	5
rbv2_xgboost	12	mfeat-factors	14
rbv2_xgboost	1501	semeion	14
rbv2_xgboost	40499	texture	14

Table S8. YAHPO-SO v1 mixed-hierarchical instances. Each row lists the scenario, instance identifier, underlying data set, and search space dimension.

Scenario	Instance	Data set	Dimension
lcbench	189873	dionis	7
lcbench	189906	segment	7
lcbench	167168	vehicle	7
nb301	CIFAR10	CIFAR10	34
rbv2_ranger	16	mfeat-karhunen	8
rbv2_ranger	42	soybean	8
rbv2_rpart	14	mfeat-fourier	5
rbv2_rpart	40499	texture	5
rbv2_super	1457	amazon-commerce-reviews	38
rbv2_super	15	breast-w	38
rbv2_super	1063	kc2	38
rbv2_xgboost	12	mfeat-factors	14
rbv2_xgboost	16	mfeat-karhunen	14
rbv2_xgboost	1501	semeion	14

Table S9. Ablation study on the numeric search space. The 'Δ start' column shows the change in RSNS when one parameter of the start configuration is changed. The 'Δ final' column shows the change in RSNS when one parameter of the final configuration is changed. Dependent parameters with unsatisfied dependencies in the start or final configuration are omitted. When a parent parameter was changed, the dependent parameters were only changed to their default values e.g. GP to RF with 500 trees and jackknife variance estimator.

Parameter	Value	Δ start	Δ final
Input trafo	Unitcube	0.00	-0.02
Output trafo	Log	0.06	–
	None	–	-0.21
	Standardize	0.00	-0.20
Init	LHS	0.01	-0.02
	Sobol	0.00	-0.01
Init size	0.05	0.05	–
	0.10	0.05	-0.03
	0.25	–	-0.01
Random interleave	2	-0.17	-0.04
	4	-0.07	-0.02
Surrogate	RF	-0.18	-0.20
Kernel	Exp	-0.07	-0.12
	Gauss	–	-0.05
	Matérn $3/2$	0.06	–
	Matérn $5/2$	0.05	-0.02
Nugget	0	–	-0.05
	10^{-3}	0.01	-0.05
	10^{-8}	0.00	–
Scaling	TRUE	0.01	-0.22
Acquisition function	CB	0.01	–
	EI	–	-0.09
	Mean	-0.02	-0.32
	PI	-0.01	-1.10
λ	1	–	-0.16
	10	–	-0.13
ϵ decay	TRUE	-0.08	–
λ decay	TRUE	–	-0.12
Optimizer	CMA-ES	0.24	–
	DIRECT	0.10	-0.11
	L-BFGS-B	0.18	-0.59
	LS	0.12	-0.01
	RS	0.02	-0.20
	RS 1000	–	-0.29

Table S10. Ablation study on the mixed-hierarchical search space. The 'Δ start' column shows the change in RSNS when one parameter of the start configuration is changed. The 'Δ final' column shows the change in RSNS when one parameter of the final configuration is changed. Dependent parameters with unsatisfied dependencies in the start or final configuration are omitted. When a parent parameter was changed, the dependent parameters were only changed to their default values e.g. EI to LCB with a λ value of 1 and no λ decay.

Parameter	Value	Δ start	Δ final
Input trafo	Unitcube	-0.01	-0.02
Output trafo	Log	0.16	–
	None	–	-0.22
	Standardize	0.02	-0.08
Init	LHS	-0.01	-0.04
	Sobol	0.00	-0.06
Init size	0.05	0.04	–
	0.10	0.04	-0.04
	0.25	–	-0.05
Random interleave	2	-0.07	-0.08
	4	-0.01	-0.03
Trees	10	-0.07	-0.15
Variance estimator	ESD	–	-0.05
	Jack	0.01	-0.08
	LTV	0.01	–
Acquisition function	CB	0.10	–
	EI	–	-0.04
	Mean	0.04	-0.12
	PI	0.09	-0.07
λ	10	–	-0.02
	3	–	-0.05
ϵ decay	TRUE	-0.22	–
λ decay	TRUE	–	-0.08
Optimizer	LS	0.10	–
	RS	0.04	-0.22
	RS 1000	–	-0.27

Note S1: Uncertainty Estimation

To estimate the uncertainty of a random forest prediction, three methods are available in `mlr3mbo`: Jackknife-after-bootstrap, ensemble variance, and the law of total variance. Let t_b be the trees in the forest, with $b \in \{1, \ldots, B\}$. Define the average of all predictors, i.e. the prediction of the random forest, to be $\bar{f}(x) = \frac{1}{B}\sum_{b=1}^{B} t_b(x)$.

For **jackknife-after-bootstrap**[S13]: Let $N_{b,i}$ be the number of times observation i occurs in-bag for tree b in the bootstrap. Then

$$f_{-i}(x) = \frac{1}{B_i} \sum_{b:N_{b,i}=0} t_b(x), \qquad B_i = |\{b : N_{b,i} = 0\}|$$

is the "leave-i-out" predictor using all trees for which observation i was out-of-bag. The predictive variance $\widehat{\sigma}^2_J(x)$ is then estimated by:

$$\widehat{\sigma}^2_J(x) = \max\left(\frac{n-1}{n}\sum_{i=1}^{n}\left(f_{-i}(x) - \bar{f}(x)\right)^2 - (e-1)\frac{n}{B^2}\sum_{b=1}^{B}\left(t_b(x) - \bar{f}(x)\right)^2, 0\right),$$

where the second sum term is a finite sample Monte Carlo bias correction.

For **ensemble variance**[S14]: Consider a fixed test observation x. For each tree t_b, compute the empirical mean μ_b of the training data points that fall into the predicted leaf node of x. This means in particular that $\mu_b = t_b(x)$ and $\bar{f}(x) = \frac{1}{B}\sum_{b=1}^{B}\mu_b$. The (naive) ensemble variance is then

$$\sigma^2_{\mathrm{ev}}(x) = \frac{1}{B-1}\sum_{b=1}^{B}\left(\mu_b - \bar{f}(x)\right)^2$$

For **law of total variance**[S14]: Again, in a similar fashion, consider a fixed x. For each tree t_b, compute the empirical mean μ_b and variance σ^2_b of the training data points that fall into the predicted leaf node of x. The variance prediction $\hat{\sigma}^2_{\mathrm{ltv}}$ is the mean of the variance σ^2_b of the leaf nodes plus the variance of the means μ_b of the leaf nodes:

$$\begin{aligned}\hat{\sigma}^2_{\mathrm{ltv}}(x) &= \frac{1}{B}\sum_{b=1}^{B}\sigma^2_b + \frac{1}{B}\sum_{b=1}^{B}\left(\mu_b - \bar{f}(x)\right)^2 \\ &= \frac{1}{B}\sum_{b=1}^{B}\left(\sigma^2_b + \mu^2_b\right) - \bar{f}(x)^2\end{aligned}$$

This captures the noise each tree sees within its leaf and the disagreements between the trees. To avoid underestimating uncertainty, sometimes a minimum variance, of e.g., 10^{-2} is assigned to leaf nodes with small σ^2_b. This approach ensures that predictions in regions with limited data reflect the inherent uncertainty.

Note S2: Output Transformations

Transformations of the target values are a common technique to improve the performance of the surrogate model [S5]. When the surrogate model is trained on the transformed target values, one would typically apply the inverse transformation to the predictive mean and standard deviation before passing them to the acquisition function, to get values on the original scale. Alternatively, predictions can be kept on the transformed scale and the acquisition function can be defined directly in that space. LogEI, for example, is explicitly designed to operate on log-transformed targets.

Log-Transformed Surrogate Models In many hyperparameter optimization scenarios, the distribution of the target variable (e.g., runtime or loss) is heavy right-tailed due to some very poorly performing configurations. These configurations can correspond to timeouts or models that fail to train and are typically assigned very large loss or runtime values. Log-transforming the target values compresses these extremes, makes the marginal distribution more symmetric and Gaussian-like, and can reduce heteroscedasticity. However, since target values are required to be positive for log transformation, it may be possible to add an offset when negative objective values can be expected. `mlr3mbo` therefore scales output values to $(0, 1)$ before applying the log transformation.

GP surrogate models tend to be more accurate in this setting when they are trained on $\log(y)$ instead of y [S5, S15], because the GP assumptions of Gaussian, homoscedastic residuals are better satisfied. Similarly, random forests tend to find more balanced splits on log-transformed targets and can perform better on heavy-tailed objectives [S16].

When LCB or PI are computed directly on the log-transformed surrogate, no inverse transformation of the predictions is required and the acquisition function is simply interpreted as operating in log-space. If, instead, these acquisition functions are to be applied on the original scale y, the predictive distribution in log-space must be mapped back to y (e.g., using the log-normal moment formulas).

LogEI Acquisition Function The LogEI acquisition function is the expected improvement on the original objective scale when the surrogate is trained on log-transformed target values [S15]. This means it is

$$\alpha_{\text{LogEI}}(\boldsymbol{x}) = \mathbb{E}\left[\max\left(f_{\min} - Y(\boldsymbol{x}), 0\right)\right],$$

with $Y(\boldsymbol{x})$ interpreted as a log-normal distribution and $\ln Y(\boldsymbol{x}) \sim \mathcal{N}(\mu(\boldsymbol{x}), \sigma^2(\boldsymbol{x}))$. The LogEI acquisition function is then defined as

$$\alpha_{\text{LogEI}}(\boldsymbol{x}) = f_{\min}\,\Phi\big(v(\boldsymbol{x})\big) - \exp\Big(\mu(\boldsymbol{x}) + \tfrac{1}{2}\sigma^2(\boldsymbol{x})\Big)\,\Phi\big(v(\boldsymbol{x}) - \sigma(\boldsymbol{x})\big), \tag{1}$$

where

$$v(\boldsymbol{x}) = \frac{\ln f_{\min} - \mu(\boldsymbol{x})}{\sigma(\boldsymbol{x})}. \tag{2}$$

Here, Φ denotes the cumulative distribution function of the standard normal distribution, and $f_{\min}$ is the best function value in the archive. Thus, LogEI computes the expected improvement on the original scale under a log-normal model, which can better capture heavy right tails than a surrogate model defined directly on y. Both the original SMAC and SMAC3 (in its SMAC4HPO facade) use LogEI as the default acquisition function whenever a log-transform of the performance values is employed [S6, S17]. LogEI should not be confused with the logarithm of the EI, which is also used by various methods [S7].

Note S3: Loop function

As an example, we show how to build a simple EGO loop function that uses the provided building blocks.

```
my_simple_ego = function(
    instance,
    surrogate,
    acq_function,
    acq_optimizer,
    init_design_size
  ) {

  # setting up the building blocks
  surrogate$archive = instance$archive # archive
  acq_function$surrogate = surrogate # surrogate model
  acq_optimizer$acq_function = acq_function # acquisition function

  search_space = instance$search_space

  # initial design
  design = generate_design_sobol(
    search_space, n = init_design_size)$data
  instance$eval_batch(design)

  # BO loop
  repeat {
    candidate = tryCatch({
      # update the surrogate model
      acq_function$surrogate$update()
      # update the acquisition function
      acq_function$update()
      # optimize the acquisition function to yield a new candidate
      acq_optimizer$optimize()
    }, mbo_error = function(mbo_error_condition) {
      generate_design_random(search_space, n = 1L)$data
    })

    # evaluate the candidate and add it to the archive
    tryCatch({
      instance$eval_batch(candidate)
    }, terminated_error = function(cond) {
      # eval_batch() throws a terminated_error if the instance is
      # already terminated, e.g. because of timeout
    })
    if (instance$is_terminated) break
  }

  return(instance)
}
```

Note S4: Local Search

The local search (LS) acquisition function optimizer in the `bbotk` package works similarly to the LS implementation in SMAC[S17] and can be used to optimize acquisition functions in mixed spaces. To reduce function call overhead, k LS runs are generated concurrently, so that as many points as possible can be passed to the SM in a single batch.

Each LS run is executed for a specified number of iterations. In each iteration, the LS generates multiple candidate neighbors by mutating the current point / incumbent. A neighbor is obtained by mutating exactly one parameter from the current point. For numeric parameters, the parameter value is first scaled to $[0, 1]$, zero-mean Gaussian noise (with standard deviation governed by a control parameter) is added, and the result is then rescaled to the original domain. Afterwards, the value is clipped to the corresponding lower and upper bounds. For integer parameters, the same procedure is applied, but the result is rounded to the nearest integer. For factor parameters, a new level is sampled uniformly from the levels that differ from the current one. For logical parameters, the value is simply flipped.

Hierarchical dependencies in the search space are handled as follows. Only active parameters are eligible for mutation. After a mutation occurs, all conditions in the search space are checked in topological order. If a condition is not satisfied, the corresponding parameter is marked as inactive. Conversely, if all conditions for a parameter are satisfied but it is marked as inactive, it is assigned a random valid value.

After the neighbors are generated, they are evaluated using the acquisition function. The number of candidates passed to the SM simultaneously is therefore k times the number of neighbors generated per LS run. For each LS, the best neighbor is selected as the new incumbent unless all neighbors are worse than the current incumbent, in which case the incumbent is retained. To avoid getting stuck in local optima, the algorithm employs a restart mechanism: For each LS, the number of consecutive iterations without improvement is tracked, and if this exceeds a specified threshold, the search is restarted from a new random point in the search space.

Note S5: Technical Details

This section provides more details on the technical settings that were not considered in the CD procedure. The function evaluation budget of the acquisition function optimization was set to $100 \cdot d^2$ evaluations. DIRECT and L-BFGS-B were randomly restarted for $5 \cdot d$ times and CMA-ES and LS were restarted until the global budget was exhausted. The random forest implementation in mlr3mbo uses the ranger package[S18] with the following settings different from the default: `sample.fraction = 1`, `min.node.size = 3`, `min.bucket = 3`, `mtry.ratio = 5/6`. The Gaussian process implementation in mlr3mbo uses the DiceKriging package[S19] with the `optim.method` changed to `"gen"`. The λ and ϵ decays reduce the value of the parameters by 1% in each iteration.

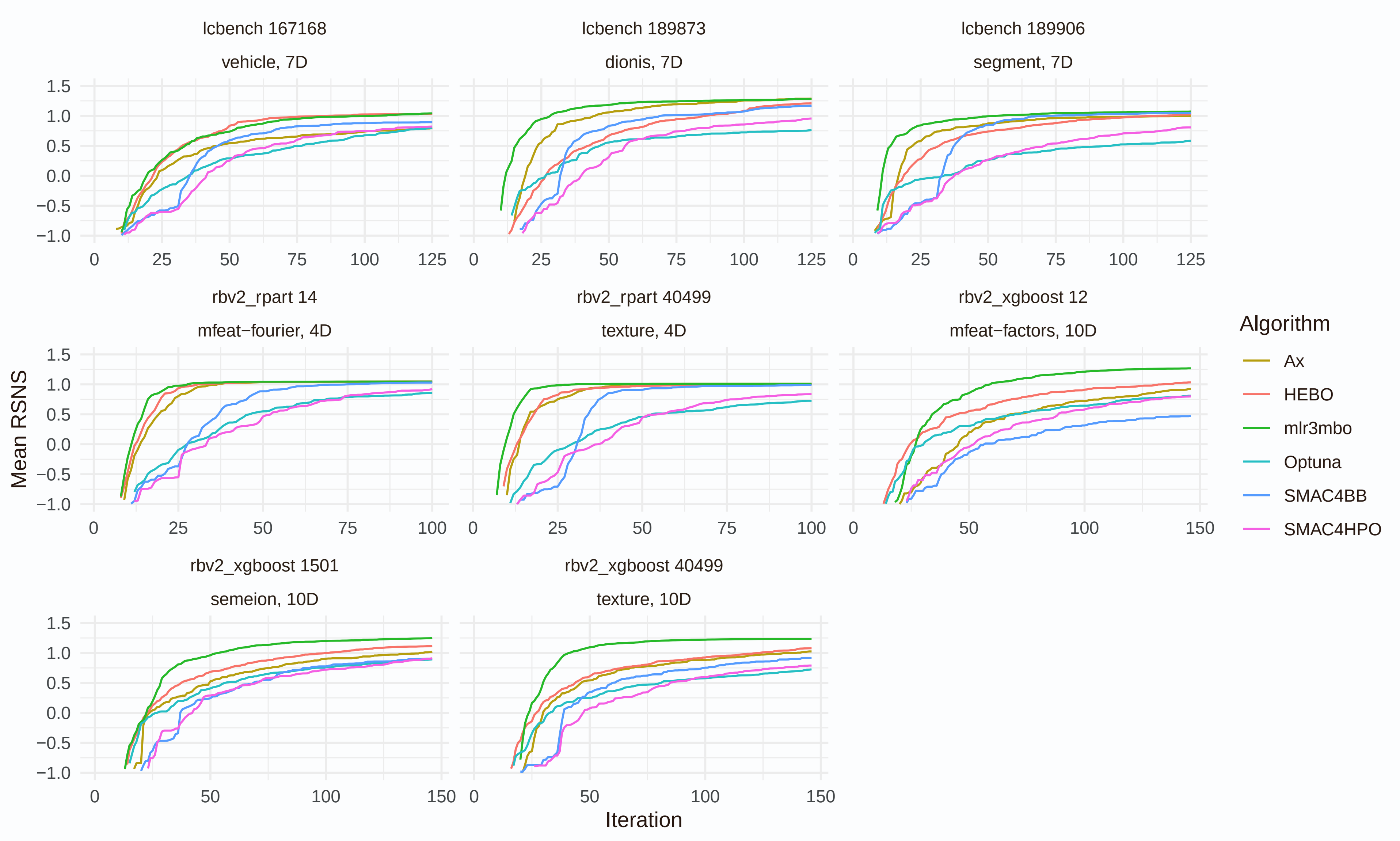

Figure S1. Optimization performance on each numeric instance. Mean RSNS as a function of iteration for `Ax`, `HEBO`, `SMAC4BB`, `SMAC4HPO`, `Optuna`, and `mlr3mbo` on each YAHPO-SO v1 numeric instance. Each panel corresponds to one instance, labeled by benchmark scenario, task ID, dataset, and search-space dimensionality. Curves are averaged over 30 runs with different random seeds and start at different iterations because the initial design size differs across frameworks. See also Figure 4A.

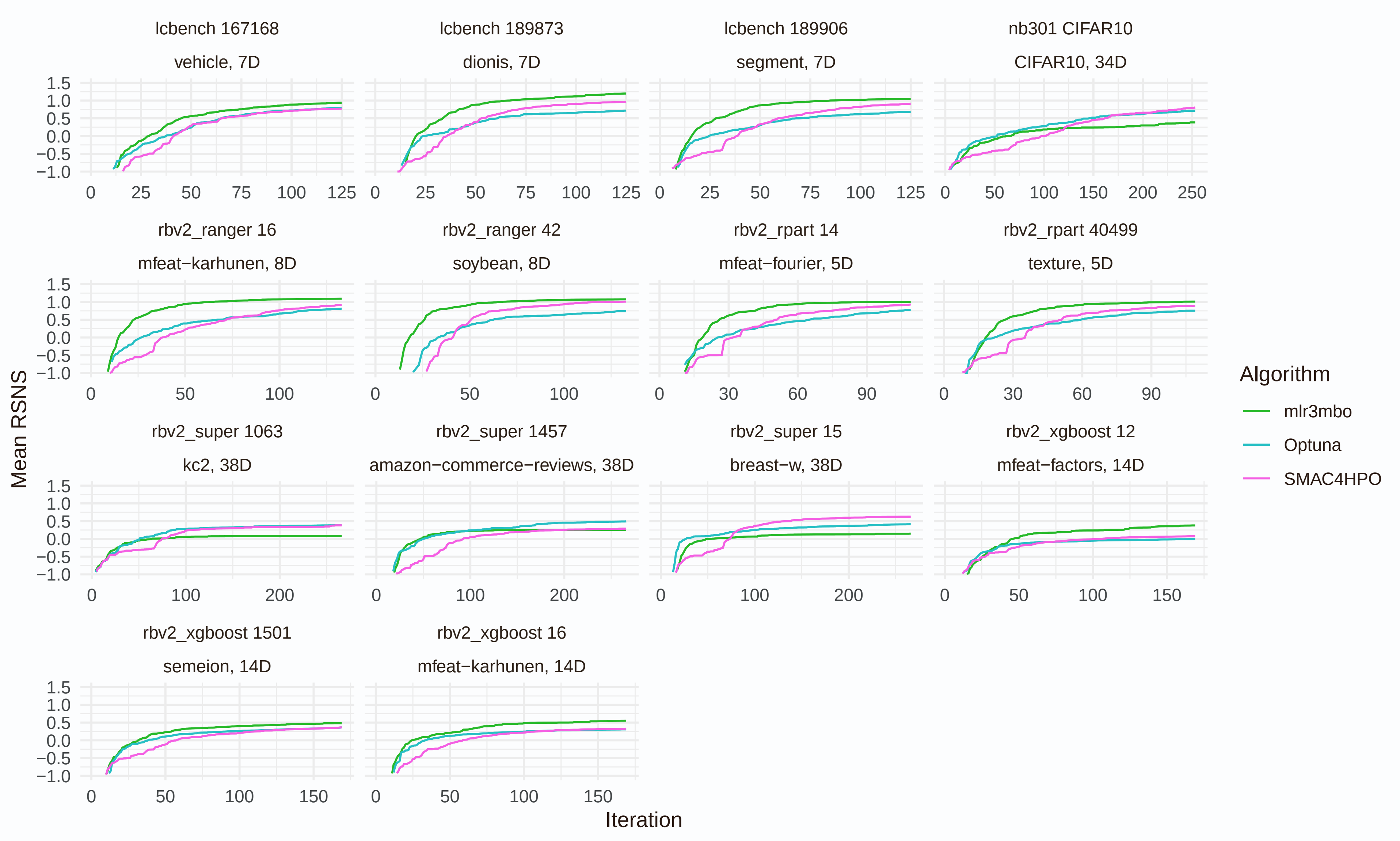

Figure S2. Optimization performance on each mixed-hierarchical instance. Mean RSNS as a function of iteration for `SMAC4HPO`, `Optuna`, and `mlr3mbo` on each YAHPO-SO v1 mixed-hierarchical instance. Each panel corresponds to one instance, labeled by benchmark scenario, task ID, dataset, and search-space dimensionality. Curves are averaged over 30 runs with different random seeds and start at different iterations because the initial design size differs across frameworks. See also Figure 4B.

References

[S1] D. Huang, T. T. Allen, W. I. Notz, and N. Zeng. Global Optimization of Stochastic Black-Box Systems via Sequential Kriging Meta-Models. *J. Glob. Optim.*, 34(3):441–466, March 2006. ISSN 1573-2916. doi: 10.1007/s10898-005-2454-3.

[S2] Jasper Snoek, Hugo Larochelle, and Ryan P. Adams. Practical Bayesian optimization of machine learning algorithms. In *Advances in Neural Information Processing Systems*, volume 25 of *NIPS'12*, pages 2951–2959. Curran Associates, Inc., December 2012.

[S3] Michael Emmerich, Kaifeng Yang, André Deutz, Hao Wang, and Carlos M. Fonseca. A Multicriteria Generalization of Bayesian Global Optimization. In Panos M. Pardalos, Anatoly Zhigljavsky, and Julius Žilinskas, editors, *Advances in Stochastic and Deterministic Global Optimization*, pages 229–242. Springer International Publishing, 2016. doi: 10.1007/978-3-319-29975-4_12.

[S4] Alma Rahat, Tinkle Chugh, Jonathan Fieldsend, Richard Allmendinger, and Kaisa Miettinen. Efficient Approximation of Expected Hypervolume Improvement Using Gauss-Hermite Quadrature. In Günter Rudolph, Anna V. Kononova, Hernán Aguirre, Pascal Kerschke, Gabriela Ochoa, and Tea Tušar, editors, *Parallel Problem Solving from Nature – PPSN XVII*, pages 90–103. Springer International Publishing, 2022. doi: 10.1007/978-3-031-14714-2_7.

[S5] Donald R. Jones, Matthias Schonlau, and William J. Welch. Efficient Global Optimization of Expensive Black-Box Functions. *J. Glob. Optim.*, 13(4):455–492, December 1998. ISSN 1573-2916. doi: 10.1023/A:1008306431147.

[S6] Frank Hutter, Holger H. Hoos, and Kevin Leyton-Brown. Sequential Model-Based Optimization for General Algorithm Configuration. In Carlos A. Coello Coello, editor, *Learning and Intelligent Optimization*, pages 507–523. Springer International Publishing, 2011. doi: 10.1007/978-3-642-25566-3_40.

[S7] Shuhei Watanabe. Derivation of Closed Form of Expected Improvement for Gaussian Process Trained on Log-Transformed Objective, November 2024.

[S8] H. J. Kushner. A New Method of Locating the Maximum Point of an Arbitrary Multipeak Curve in the Presence of Noise. *J. Basic Eng.*, 86(1):97–106, March 1964. ISSN 0021-9223. doi: 10.1115/1.3653121.

[S9] Nicola Beume, Boris Naujoks, and Michael Emmerich. SMS-EMOA: Multiobjective selection based on dominated hypervolume. *Eur. J. Oper. Res.*, 181(3):1653–1669, September 2007. ISSN 0377-2217. doi: 10.1016/j.ejor.2006.08.008.

[S10] D. R. Jones, C. D. Perttunen, and B. E. Stuckman. Lipschitzian optimization without the Lipschitz constant. *J. Optim. Theory Appl.*, 79(1):157–181, October 1993. ISSN 1573-2878. doi: 10.1007/BF00941892.

[S11] Richard H. Byrd, Peihuang Lu, Jorge Nocedal, and Ciyou Zhu. A Limited Memory Algorithm for Bound Constrained Optimization. *SIAM J. Sci. Comput.*, 16(5):1190–1208, September 1995. ISSN 1064-8275. doi: 10.1137/0916069.

[S12] Nikolaus Hansen and Andreas Ostermeier. Completely Derandomized Self-Adaptation in Evolution Strategies. *Evol. Comput.*, 9(2):159–195, June 2001. ISSN 1063-6560. doi: 10.1162/106365601750190398.

[S13] Stefan Wager, Trevor Hastie, and Bradley Efron. Confidence intervals for random forests: The jackknife and the infinitesimal jackknife. *J. Mach. Learn. Res.*, 15(1):1625–1651, January 2014. ISSN 1532-4435.

[S14] Frank Hutter, Lin Xu, Holger H. Hoos, and Kevin Leyton-Brown. Algorithm runtime prediction: Methods & evaluation. *Artif. Intell.*, 206:79–111, January 2014. ISSN 00043702. doi: 10.1016/j.artint.2013.10.003.

[S15] Frank Hutter, Holger H. Hoos, Kevin Leyton-Brown, and Kevin P. Murphy. An experimental investigation of model-based parameter optimisation: SPO and beyond. In *Proceedings of the 11th Annual Conference on Genetic and Evolutionary Computation*, GECCO '09, pages 271–278. Association for Computing Machinery, July 2009. doi: 10.1145/1569901.1569940.

[S16] Marius Lindauer, Matthias Feurer, Katharina Eggensperger, André Biedenkapp, and Frank Hutter. Towards Assessing the Impact of Bayesian Optimization's Own Hyperparameters, August 2019.

[S17] Marius Lindauer, Katharina Eggensperger, Matthias Feurer, André Biedenkapp, Difan Deng, Carolin Benjamins, Tim Ruhkopf, René Sass, and Frank Hutter. SMAC3: A Versatile Bayesian Optimization Package for Hyperparameter Optimization. *J. Mach. Learn. Res.*, 23(54):1–9, 2022. ISSN 1533-7928.

[S18] Marvin N. Wright and Andreas Ziegler. Ranger: A Fast Implementation of Random Forests for High Dimensional Data in C++ and R. *J. Stat. Softw.*, 77(1):1–17, 2017. ISSN 1548-7660. doi: 10.18637/jss.v077.i01.

[S19] Olivier Roustant, David Ginsbourger, and Yves Deville. DiceKriging, DiceOptim: Two R packages for the analysis of computer experiments by kriging-based metamodeling and optimization. *J. Stat. Softw.*, 51(1):1–55, 2012. doi: 10.18637/jss.v051.i01.
